# Predictors and evolution of post-stroke depression among adults admitted for first stroke at referral hospitals in Dodoma, Tanzania: A protocol for a prospective longitudinal observational study

**DOI:** 10.1371/journal.pone.0352784

**Published:** 2026-07-22

**Authors:** Sadiki Mandari, Azan Nyundo

**Affiliations:** 1 Department of Psychiatry and Mental Health, School of Medicine, The University of Dodoma, Dodoma, Tanzania; 2 Mirembe National Mental Health Hospital, Dodoma, Tanzania; 3 Department of Internal Medicine, The Benjamin Mkapa Hospital, Dodoma, Tanzania; Muhimbili National Hospital, TANZANIA, UNITED REPUBLIC OF

## Abstract

**Background:**

Survivors of strokes are prone to disabilities, especially in underdeveloped countries. Post-stroke depression (PSD) is a common neuropsychiatric condition that exacerbates symptoms and raises the danger of stroke recurrence, disability, and mortality. Nevertheless, little is documented about PSD’s incidence, predictors, and consequences.

This study aims to assess predictors and outcomes of post-stroke depression among patients admitted with the first stroke episode at referral hospitals in Dodoma, Tanzania.

**Methods and analysis:**

The study is a prospective longitudinal observational design; a consecutive sampling technique will be used to attain the estimated sample size of adults aged ≥18 years who have had their first stroke episode, within 14 days. The study will be conducted at referral hospitals in the Dodoma region, Tanzania. At admission, baseline clinical and sociodemographic parameters will be recorded, including relevant medical and psychiatric history. Depressive symptoms will be assessed at 1- and 3-months post-stroke, with the 1-month assessment serving as the baseline for post-stroke depression. The International Neuropsychiatric Interview (M.I.N.I) will be used to diagnose major depressive disorder and to exclude participants with a prior history of depression, while the PHQ-9 will be used to quantify severity and monitor progression of depressive symptoms over time. Data will be summarised using descriptive statistics; continuous data will be reported as mean (SD) or median (IQR), while categorical data will be reported as frequencies and proportions. The PSD predictors will be determined using logistic regression analysis. The study will adhere to data-sharing guidelines and take ethical considerations into account.

**Ethics and dissemination:**

The University of Dodoma’s institutional Research Review and Ethical Committee has granted permission to conduct the study with reference number MA.84/261/02. The relevant authorities granted approval for the study to be carried out at DRRH and BMH.

## Introduction

Stroke is the second leading cause of death and a third leading cause of death and disability combined [1] and is also linked to the development of psychiatric symptoms and impaired quality of life [2]. Similar to high-income settings, where the majority of stroke survivors have Ischaemic stroke, approximately 80% of strokes in developing countries are also Ischaemic [3,4]. However, emerging evidence suggests a shift toward a higher relative contribution of haemorrhagic stroke in some sub-Saharan African settings. For instance, a recent multi-centre study in Tanzania reported a higher prevalence of haemorrhagic stroke (58%) compared to ischaemic stroke (42%) [5]. This pattern reflects context-specific factors, including referral bias to tertiary centres where more severe haemorrhagic cases predominate, the inclusion of specialized facilities such as Muhimbili Orthopaedic Institute (primarily admits haemorrhagic stroke), and the high burden of uncontrolled hypertension and delayed presentation.

Sub-Saharan Africa and other low and middle-income countries, including Tanzania are disproportionally over represented with the burden of stroke. Between1990–2019, stroke incidence increased by 70% with narked rise in overall disease burden [6]. Despite the implementation of preventive measures and risk factor reduction similar to high-income countries, a high rates of stroke continue to be recorded in both urban and rural areas of Tanzania [7]. The risk of Following neuropsychiatric disorders particularly post-stroke depression increases substantially following a stroke, which is also associated with significant increases in mortality rates, a one-month PSD incidence of 20–50% is reported and may persist for six months or longer after the stroke [8,9]**.** Studies from high-income countries have reported post-stroke depression prevalence estimates ranging from 25% to 79%, reflecting substantial methodological and population heterogeneity [8,10]*.* The pooled prevalence of post-stroke depression in Africa is high, estimated at 38.35% (95% CI: 34.07–42.63%), with comparable estimates obtained using both clinical diagnostic tools and rating scales. Notably, regional variation exists, with substantially higher prevalence observed in Central Africa (50.92%, 95% CI: 45.94–55.90%), highlighting potential contextual and health system influences [11]. In Sub-Saharan Africa, evidence remains limited and heterogeneous. In Tanzania, the MAMBO trial screened post-stroke outcomes, including depressive symptoms using the PHQ-9; however, the focus was primarily on motor recovery and did not evaluate the incidence of clinically defined depression. This highlights a critical gap in the literature regarding the burden and characterization of PSD in this setting [12].

Although there is mixed evidence, several factors, including female gender, advanced age, medical and psychiatric history, social support, and stroke-related factors, including severity and degree of disability, have been accounted for in PSD. [13].

The study aims to determine the incidence, predictors and outcome of PSD among patients admitted with the first stroke episode at referral hospitals in Dodoma, Tanzania.

### Study aims

#### Aim 1.

To determine the incidence of post-stroke depression at one month among patients admitted with the first stroke episode at referral Hospitals in Dodoma.

#### Aim 2.

To determine predictors of post-stroke depression among patients admitted with the first stroke episode at referral Hospitals in Dodoma.

#### Aim 3.

To determine the outcome of post-stroke depressive symptoms at three months following the first stroke episode among patients admitted at referral Hospitals in Dodoma.

#### Aim 4.

To determine predictors of the evolution of depressive symptoms among patients admitted with the first episode of a stroke at referral Hospitals in Dodoma.

## Methods and analysis

### Study design

The study will be a prospective longitudinal observation design.

### Study setting

The study will be conducted in Dodoma’s referral hospitals, the Dodoma Regional Referral Hospital (DRRH) and Benjamin Mkapa Hospital (BMH). Dodoma is Tanzania’s capital, with a population of 3,085,625 people per the 2022 national census [14]. The coverage includes referrals from all Dodoma districts of Mpwapwa, Bahi, Kongwa, Chemba, Kondoa, and neighbouring regions. BMH has 400 beds while DRRH has 480 bed capacities, where all patients will be received at the emergency department before being transferred to the respective unit or ward. Stroke is among the top listed conditions admitted at the hospitals, with about 20 cases admitted every month in the medical ward and intensive care unit for those requiring close observation. Also, both BMH and DRRH have advanced radiological investigations, including CT-scan and MRI covering patients from neighbouring regions. Patients are typically referred from primary and secondary health facilities, including district hospitals and health centres, although some may present directly to these referral hospitals. Delays in presentation may occur due to factors such as transport challenges, referral system inefficiencies, limited awareness of stroke symptoms, and financial barriers, which may result in underrepresentation of stroke cases within the hospital-based sample.

### Study population

Participants are adults aged 18 years or older admitted to the Internal Medicine wards of either DRRH or BMH with a diagnosis of the first stroke as per the World Health Organization, defined as “rapid development of clinical signs of focal or global disturbance of cerebral function lasting more than 24 hours or leading to death, with no apparent cause other than vascular origin” [15] For the purpose of this study, two additional operational criteria will be applied: (1) presentation within 14 days of symptom onset to ensure recruitment within the acute phase, and (2) radiological confirmation of stroke using computed tomography (CT) or magnetic resonance imaging (MRI). Neuroimaging will also help in excluding transient ischaemic attack and classify stroke subtype (ischaemic or haemorrhagic). Patients with symptoms resolving within 24 hours without imaging evidence of acute infarction or haemorrhage will be excluded as TIA.

### Inclusion criteria

- Patients aged 18 years or older admitted with the first episode of stroke.
- Capacity to provide informed consent or proxy consent from a close relative or custodian in case the patient is incapable.
- Patients with the first stroke episode within 14 days confirmed by CT-scan/MRI.

### Exclusion criteria

- All patients with severe sensory impairment (deafness and blindness) will compromise the assessment of key dependent and independent variables.
- Patients with traumatic intracerebral haemorrhage.
- Patients with intracerebral haemorrhage due to tumour.
- Patients with transient ischemic attack (TIA).
- Patients with traumatic subarachnoid haemorrhage. Patients with a known history of chronic neurological disorders with an established risk of psychiatric manifestations, such as epilepsy, multiple sclerosis, and neurodegenerative disorders.

### Sample size calculation

The sample size for estimating the prevalence of post-stroke depression was calculated using the finite population correction to the single population proportion formula described by Cochran and Kish. Assuming an expected prevalence of 30%, 95% confidence level, and 5% margin of error, the corrected sample size was computed as n = [N × Z² × p(1-p)] / [d²(N-1) + Z² × p(1-p)] [16–18]. After adjustment for an anticipated 30% loss to follow-up, the minimum required sample size is 312 participants if the available source population was 500.

Where:

n = required sample size before adjustment for attrition

N = finite population size

Z = standard normal deviate for the desired confidence level (1.96 for 95% confidence)

p = expected prevalence of post-stroke depression

1-p = proportion without post-stroke depression

d = margin of error (precision)

Adjustment for loss to follow-up:

n_final (N) = n / (1-r)

Where r is the anticipated attrition proportion. In this study, r = 0.30.

Assumptions used

Expected prevalence of post-stroke depression at 1 month: p = 0.30

Confidence level: 95% (Z = 1.96)

Margin of error: d = 0.05

Anticipated attrition by 3 months: 30% (r = 0.30)

Given that the maximum expected available population is 500 over 12 months period,

Substituting N = 500 into the finite population correction formula:

n = [500 × 1.96² × 0.30 × 0.70] / [0.05² (499) + 1.96² × 0.30 × 0.70]

This gives n = 218 participants before adjustment for attrition.

N final = 218 / 0.70 = 312

Therefore, if the maximum source population is 500, the corrected minimum recruitment target is 312 participants.

### Sampling methods/technique

A consecutive sampling method will be used, whereby the sample will be attained by selecting every available candidate meeting the inclusion criteria and admitted through the emergency/outpatient department to the wards until the desired sample size is reached.

### Data collection procedure/recruitment of patients

At baseline: direct interviews with the patient and/or their immediate guardian/custodian will be conducted to collect socio-demographic information (including sex, age, marital status, and occupation). Detailed history and physical examination will be performed to identify vascular risk factors, such as hypertension, diabetes mellitus, dyslipidaemia, lifestyle factors smoking, alcohol use (defined as any use within the past 12 months.), prior stroke or transient ischemic attack, and cardiac history. Neurological examination will be conducted to document deficits and localize the lesion.

A comprehensive clinical assessment will be performed, including detailed history taking and physical examination.

Blood pressure (BP) readings will be taken using an automated digital machine AD Medical Inc. brand; patient will be in a supine position with the arm placed at the same position as the heart; a minimum of two readings will be taken 2 minutes apart. The affected arm will be avoided to reduce false results. Hypertension will be defined as BP ≥ 140/90 mmHg in patients with a history of hypertension or on antihypertensive medications [19]. Oxygen saturation SpO2 will be assessed using pulse oximetry. Atrial fibrillation, other arrhythmias and pulse rare will be recorded from electrocardiography (ECG).

All participants will undergo brain imaging using computed tomography (CT) or magnetic resonance imaging (MRI) to confirm the diagnosis of stroke, differentiate between ischemic and haemorrhagic stroke, and characterize lesion location, size, and laterality. Imaging will also be used to assess features such as Leukoaraiosis and other structural abnormalities.

Stroke severity will be assessed using the National Institutes of Health Stroke Scale (NIHSS), which has a maximum score of 42. Stroke severity will be categorized as follows: mild (NIHSS 1–4), moderate (5–15), severe (16–20), and very severe (21–42) [20]. Patients who will score ≤5 usually indicate a strong possibility for a good recovery, with a sensitivity of 72% and specificity of 89% [21].

### Laboratory investigations

This study’s testing will be done in accordance with accepted DRRH and BMH standard operating procedures. A trained laboratory technologist will obtain informed consent from each participant prior to venepuncture and finger-prick procedures. Before sample collection, appropriately labelled blood collection tubes EDTA (K2/K3), sodium fluoride, and plain (serum) tubes will be prepared and labelled with the participant’s hospital registration number.

A tourniquet will be applied approximately 5–7 cm above the antecubital fossa, and the puncture site will be disinfected using 70% methylated spirit in a circular motion, followed by air drying for approximately 20 seconds. Using aseptic technique, 5 mL of venous blood will be drawn from one arm using a sterile syringe and transferred into appropriate tubes.

Following blood collection, the tourniquet will be released, and haemostasis will be achieved by applying gentle pressure with a sterile cotton swab.

Samples will be transported in a cool box (2-8°C) to the laboratories at Dodoma Regional Referral Hospital and Benjamin Mkapa Hospital within 3 hours of collection. Haemolysed samples will be discarded, and repeat venepuncture will be performed where necessary.

For serum separation, blood in plain tubes will be allowed to clot and then centrifuged at 3000 rpm for 5–10 minutes (not 300 rpm, which is insufficient). The resulting serum will be aliquoted into two portions: one for lipid profile analysis and the other for serum electrolytes.

If analysis is delayed beyond 2 hours, serum samples will be stored at −20°C to 80°C. If analysis is performed within 2 hours, samples will be kept at room temperature (20–25°C). Biochemical analysis will be conducted using an automated clinical chemistry analyser (Elba XL-180, Germany; Serial No. 160239) following manufacturer protocols. A high total cholesterol level of 200 mg/dL or higher, a low-density lipoprotein cholesterol level of 160 mg/dL or higher, a triglyceride level of 150 mg/dL or higher, or a high-density lipoprotein cholesterol level of 40 mg/dL for women and 50 mg/dL for males were all considered to be signs of dyslipidaemia [22].

Capillary blood glucose measurement will be performed to assess current glycaemic status and identify hyperglycaemia and undiagnosed diabetes mellitus among participants.

The participant’s hand will be positioned palm facing upward, and the lateral aspect of the fingertip (preferably the middle or ring finger) will be selected. The finger will be gently massaged to enhance blood flow, avoiding excessive squeezing to prevent haemodilution. The puncture site will be cleansed using 70% isopropyl alcohol (or methylated spirit) and allowed to air dry completely for approximately 20–30 seconds.

The finger will then be held slightly below the level of the elbow to facilitate blood flow and pricked using a single-use sterile lancet. The first drop of blood will be wiped away with sterile gauze, and a subsequent drop will be applied directly onto a test strip inserted into the Accu-Chek Active glucometer (Roche Diagnostics) for immediate measurement of blood glucose.

The procedure will follow the Sample Collection Manual (SM-1–03.3) used at Benjamin Mkapa Hospital and Dodoma Regional Referral Hospital. After sample collection, gentle pressure will be applied to the puncture site using a sterile cotton swab until bleeding stops (typically 1–2 minutes). The used lancet will be immediately discarded into an approved sharps disposal container.

Hyperglycaemia will be defined according to the American Diabetes Association [23] For non-diabetic patients, hyperglycaemia will be defined as random blood sugar >11.1 mmol/L, or fasting blood sugar > 7.0 mmol/L, and a diagnosis of diabetes will be made with a fasting blood sugar ≥ 7.0 mmol/L, or random blood glucose ≥ 11.1 mmo/L plus symptoms of hyperglycaemia or glycated haemoglobin ≥ 6.5%. HbA1c will further be categorized as normal (< 5.7%), prediabetes (5.7%–6.4%), and diabetes (≥ 6.5%), and will be used to assess chronic glycaemic status and to distinguish stress hyperglycaemia from underlying dysglycaemia in participants presenting with elevated random blood glucose levels.

To ensure the validity and reliability of laboratory results, internal quality control procedures will be performed daily at the BMH and the DRRH. Essential maintenance is performed once every six months, whereas once a week is reserved for machine maintenance.

### Electrocardiogram (ECG)

A 12-lead ECG will be carried out with the manufacturer’s guidelines [24] under the direction of a professional cardiologist for each participant. Before a 12-lead ECG is taken, the patient will be informed about the procedure, their privacy will be protected, and the environment will be kept comfortable to help the patient feel at ease and prevent interference with the ECG trace’s clarity. The necessary tools, such as the electrocardiograph, ECG paper, and ECG tabs, will be available to attach the electrodes and leads to the patient. The ECG cables must be kept from being twisted to prevent interference with ECG tracing. The patient will be directed to lie down at an angle of 45 degrees with his or her head properly supported and the bed’s backrest, with the inner aspect of the patient’s wrist close to but not touching the patient’s waist. This is done after entering the patient’s ID number into the device and getting consent. As long as wet gel electrodes are utilised, shaving the skin won’t be necessary. The limb electrodes will next be placed in the following manner: Right inner wrist is red, left inner wrist is yellow, right inner leg is black just above the ankle, and left inner leg is green just above the ankle. The chest leads will be organised as follows: V4 is in the fifth intercostal space, mid-clavicular line, V5 is in the anterior axillary line, and V6 is in the mid-axillary line, the same horizontal line as V4 and V6. V1 is immediately to the right of the sternum, V2 is immediately to the left of the sternum, V3 is halfway between V4 and V2, V4 is in the fifth intercostal space, mid-clavicular line, and V6 is in the mid-axillary line, the same horizontal line as V4 and V5. ECG cables should not lie to each other and tension should be avoided to decrease artefact and increase the accuracy and quality of ECG tracing. The calibration signal on the ECG machine should be kept at a paper speed of 25 millimetre/second and ECG size 1 millivolt/10-millimeter deflection. During the procedure, the patient will be asked to remain motionless and breathe normally; the ECG trace should be clear prior to recording. The 12-lead ECG trace will include the patient’s name, hospital identification number, date of birth, and the day and time the ECG was taken. According to the American College of Cardiology’s management guidelines for atrial fibrillation patients, the absence of P waves and an irregular-irregular RR interval are diagnostic signs of the condition [25–27].

### Echocardiography

Only certain patients with ischemic stroke and additional characteristics, such as evidence of cardiac disease on history, examination, or electrocardiogram (ECG), suspected cardiac source of embolism (for example, infarctions in multiple cerebral or systemic arterial territories), suspected aortic disease, or paradoxical embolism, as well as patients with no other options, will be advised to undergo transthoracic echocardiography (model Vivid TM T9 made by GE Healthcare, USA, 2018)**.**

### Brain imaging

To confirm the stroke diagnosis, every patient will have an acute CT scan by SIEMENS (SOMATOM Definition Flash), and the majority will also get a brain MRI scan by MAGNETUM SPECTRA A TIM + Dot System 3T as part of a standard diagnostic procedure. Within the first 14 days following a stroke, patients with stroke-like symptoms but negative haemorrhagic stroke CT scan and unknown ischemic stroke status will be recruited for a study-specific MRI brain scan. Standard MRI brain sequences for stroke axial diffusion-weighted imaging (DWI), apparent diffusion coefficient (ADC), fluid-attenuated inversion recovery (FLAIR), gradient-recalled echo (GRE), T1-weighted (T1W), and T2-weighted (T2W) will be captured for all participants undergoing MRI, as these sequences are essential for the characterization of acute and chronic cerebrovascular lesions [24,25].

Additional sequences may be performed where clinically indicated. These may include susceptibility-weighted imaging (SWI) for enhanced detection of microbleeds and haemorrhage [28], perfusion-weighted imaging (PWI) for evaluation of ischaemic penumbra [24,27], and MR angiography (MRA) for assessment of intracranial and extracranial vessels (e.g., stenosis, occlusion, or vascular malformations) [29,30]. These sequences will be used selectively based on clinical indication and research needs.

MR Brain Angiography (MRA) will be performed in selected patients where vascular pathology is suspected or where further characterization of stroke aetiology is required, rather than routinely in all participants [25,27].

MRI examinations will be conducted using 3 Tesla (3T) MRI scanners available at the study sites, ensuring high-resolution imaging and improved sensitivity for detecting acute infarcts, small vessel disease, and microbleeds [24,31].

For patients who undergo CT brain imaging, MRI will not be performed routinely. CT will serve as the initial imaging modality, particularly in the acute setting. MRI will be performed selectively based on clinical indications, including unclear diagnosis after CT, suspected early or posterior circulation stroke, need for detailed lesion characterization, or specific research requirements [29].

Neuroimaging studies will be used to differentiate ischaemic from haemorrhagic stroke, quantify haematoma volume (cm³) in haemorrhagic cases, identify the likely stroke aetiology, and determine lesion location and vascular territory, as well as to assess infarct extent using the ASPECTS score in ischaemic stroke [32]. Ischaemic stroke aetiology will be classified using the TOAST criteria [33], while haemorrhagic stroke will be classified into primary intracerebral haemorrhage (e.g., hypertensive or cerebral amyloid angiopathy-related) and secondary intracerebral haemorrhage (e.g., due to vascular malformations, aneurysms, tumours, or coagulopathies) based on clinical and imaging findings [34] 2022 AHA/ASA Guideline for the Management of Spontaneous Intracerebral Hemorrhage.

### Study variables and measures

**Aim 1 Study Variables:** Post-stroke depression at one month will be diagnosed using the Mini International Neuropsychiatric Interview (M.I.N.I), which will serve as the diagnostic reference standard for major depressive disorder (MDD). Depressive symptom severity and changes over time will be assessed using the PHQ-9 at one month and three months. The PHQ-9 is included because it provides a continuous symptom severity score, whereas the MINI primarily provides a categorical diagnosis of MDD, indicating whether diagnostic criteria are met or not. (See Table 1 for a list of the variables with a description of aim 1).

**Aim 2 Study Variables:** The variables address the predictors of post-stroke depression at one month; these include age (in years), sex, alcohol use, cigarette smoking history, history of diabetes mellitus, dyslipidaemia, atrial fibrillation, post-stroke cognitive impairment and apathy, quality of life, stroke type and characteristic (haemorrhagic/ischaemic, cortical/sub-cortical), stroke (infarct/hematoma) volume, presence of Leukoaraiosis or brain atrophy (See Table 2 for a list of the variables with a concise description of Aim 2)

**Aim 3 Study Variables:** The variables address the evolution of post-stroke depressive symptoms at three months, categorised as either significant improvement, significant worsening, or without significant change (See Table 3 for a list of the variables with a concise explanation for Aim 3).

**Aim 4 Study Variables:** The variables address the predictors of the evolution of depressive symptoms at three months (See Table 4 for a list of the variables with a concise explanation for Aim 4).

#### Dependent variables.

**Primary dependent variable**: Post-stroke depression:

The diagnosis of post-stroke depression will be established using the Mini interview based on DSM-5 criteria. Participants will be categorized as either meeting or not meeting the criteria for Major Depressive Disorder. The severity of depressive symptoms and changes in symptom burden over time will be evaluated using the Patient Health Questionnaire-9 (PHQ-9), with repeated assessments conducted during follow-up.[35].

**Secondary dependent variable:**The outcome of PSD at three months will be evaluated by change of the PHQ-9 scores, categorised as a significant improvement if scores decrease by at least 5 points, significant worsening if scores increase by at least 5 points, and no significant change if the scores remain within 5 points [36,37].

### Assessment of change in Depressive symptoms

Patient Health Questionnaire (PHQ-9) will be used to assess the progress of depressive symptoms from baseline and after the three months of follow-up. The tool has a total score of 27, using the following nine items: little interest or pleasure in doing things, feeling down, depressed, or hopeless, trouble falling or staying asleep, or sleeping too much, feeling tired or having little energy, poor appetite or overeating, feeling bad about yourself or that you are a failure or have let yourself or your family down, trouble concentrating on things, such as reading the newspaper or watching television, moving or speaking so slowly that other people could have noticed and thoughts that you would be better off dead or of hurting yourself. Each item can score from 0−3, 0 if the participant replies (not at all), 1 if the participant responds (several days), 2 if responds (more than half the days), and 3 if participant responds (nearly every day). A final total score from each item is categorised as follows: a score of 1−4 will be regarded as minimal depression, 5–9 as mild depression, 10–14 as moderate depression, 15–19 as moderately severe depression, and 20−27 as severe depression. PHQ-9 has been validated in Tanzania with a Sensitivity of 78% and a Specificity of 87% [38].

#### Independent variables.

Age (in years), sex, marital status, occupation, level of education, alcohol use, cigarette smoking history, diabetes mellitus, hypertension, dyslipidaemia, atrial fibrillation, type of stroke, lesion location, severity of stroke, stroke (infarct/hematoma) volume, presence of Leukoaraiosis, post-stroke cognitive impairment and apathy and quality of life.

### Assessment of Neurocognitive Functioning

The cognitive impairment will also be assessed at one month using the Montreal Cognitive Assessment (MoCA), which is used to evaluate cognitive impairment, with the following domains: visuospatial/executive function(score of 5), naming(score of 3), attention(score of 6), language(repeat(score of 2) and fluency(score of 1), abstraction(score of 2), delayed recall(score of 5) and orientation(score of 6). Where the optimal cut-off score point will be at a score of 22, with a sensitivity of 80% and specificity of 74%, and dementia at a score of 16, giving a sensitivity of 90% and specificity of 80% according to the validation of the tool done in Tanzania of which used the MoCA-5-minute [39].

### Assessment of Apathy

Apathy will also be assessed using an evaluation scale that provides for behavioural, emotional, and cognitive aspects of apathy generating a continuous score that allows objective quantification of symptom severity [36,37]. The tool comprises 18 items with a cut-off score of 39–41, with a good sensitivity and specificity ranging from 70–85%, supporting its use as both a screening and severity assessment tool in neurological populations [40–43].

**Assessment of functional independency, disabilities and stroke severity Barthel Index (BI)**: The functional independency in activities of daily living (ADLs) will be assessed using The Barthel Index which has been demonstrated to be suitable for acute and sub-acute of stroke. The tool assesses basic core deficits of ADLs in early and sub-acute phases after stroke ADLs, it is scored across 10 basic activities of daily living, with each item assigned a weighted score based on the level of independence (e.g., 0 = dependent, intermediate scores = partial assistance, maximum score = independent). The individual item scores are summed to give a total ranging from 0 to 100, where higher scores indicate greater functional independence. Scores are commonly interpreted as 0–20 (total dependency), 21–60 (severe dependency), 61–90 (moderate dependency), and 91–100 (slight dependency to independence). The initial level of dependency following the acute stroke event will be established as baseline, and compared to subsequent recovery and enabling adjustment for baseline disability when examining outcomes such as post-stroke depression. At 1- and 3-month follow-up, the BI will be repeated to measure functional recovery over time and detect changes in independence, allowing correlation with depressive symptom trajectories and comparison with global disability using the Modified Rankin Scale. The Barthel Index is particularly suitable for this study due to its simplicity, strong reliability, responsiveness, and focus on patient-centred functional outcomes; it complements neurological severity measures such as the NIH Stroke Scale, enabling a comprehensive assessment of recovery. Importantly, its applicability in the Tanzanian context has been demonstrated in stroke cohort studies, where functional disability measured using the Barthel Index was a significant predictor of long-term outcomes including mortality, supporting its clinical and prognostic validity in this setting [44–46].

### Modiefied Ranking Scale

The Modified Rankin Scale (mRS) will be used to assess global disability and dependence in combination with the Barthel Index. The mRS is widely regarded as the gold standard functional outcome measure in stroke trials and is extensively used in studies of the acute and sub-acute phases of stroke to evaluate functional outcomes, including rehabilitation and neuropsychiatric sequelae [47,48]. Although not a diagnostic instrument, the mRS demonstrates good validity and inter-rater reliability, with weighted κ values typically ranging from 0.70 to 0.90, and shows good correlation with other established measures such as the National Institutes of Health Stroke Scale and the Barthel Index [47]. It is also well suited for categorising functional independence and dependence, particularly when dichotomised (e.g., scores 0–2 versus 3–6) in both clinical and research settings [48].

The Barthel Index will complement the mRS by providing a more granular and objective assessment of activities of daily living (ADLs). It is a widely validated tool in stroke rehabilitation research, demonstrating good reliability, validity, and responsiveness to change over time, making it particularly suitable for assessing functional recovery and monitoring rehabilitation outcomes [49].

### National Institutes of Health Stroke Scale (NIHSS)

Baseline stroke severity will be assessed at admission using the National Institutes of Health Stroke Scale (NIHSS). The NIHSS is a structured clinical tool used to quantify the severity of neurological deficits following a stroke. It assesses key domains including level of consciousness, language, motor function, sensory function, visual fields, coordination, and attention. Scores range from 0 to 42, with higher scores indicating more severe impairment.

The NIHSS is widely used in both clinical practice and research, particularly in the acute phase of stroke, and has been shown to have good reliability and validity. It is also a useful predictor of functional outcomes and mortality following stroke [29,48,50].

### Data analysis

All data collected will be coded and entered the computer for analysis. Data will be analysed using the Statistical Package for Social Sciences (SPSS) version 25.0. The data will be described by frequencies, proportions, Mean (± SD), and Median (IQR). The Chi-square test will be used to compare the association between post-stroke depression and the independent categorical predictors. To determine the association between the independent variables and the post-stroke depression at one month and the outcome of depressive symptoms at three months, binary logistic regression analysis will be used. Multivariable models will include age and sex as covariates, along with other variables showing p < 0.2 in the univariate analyses, to control for potential confounding. Paired t-tests will be used to compare the mean changes in depressive symptoms from baseline to three months. Also, an adjusted odds ratio (aOR) with a confidence interval of 95%, set at a significance level of <0.05 will represent the results.

### Ethical approval and data dissemination

The ethical clearance was obtained from the institutional Research review committee of The University of Dodoma with the reference number MA.84/261/09. Permission to conduct the study was provided by the administration of Dodoma Regional Referral Hospital (PB.22/1307/02/114) and Benjamin Mkapa Hospital (AB/150/293/01/391). All study participants will be required to sign written informed consent forms or proxy consent from a close relative or custodian in case the patient is incapable, which will clearly state about the study. For confidentiality, participant names will not be utilised; only numbers will be used. Patients with the need for psychiatric including those with post-stroke depression, will be referred for further evaluation and management. Neuroimaging (CT or MRI) will be mandatory for study eligibility; however, no participant will incur any cost for these investigations, as the study will support all required imaging procedures.

### Study timeline

The study will be conducted for 18 months from July 2026 to October 2027. Participant recruitment will be completed on June 2027and, and overall data collection will be completed at July 2028 and results will be available by August 2027.

## Discussion

The prospective observation longitudinal nature of the study offers a robust temporal association of the prevalence, progression, and associated factors of post-stroke depression.

The PHQ-9 screening tool is highly sensitive and specific in assessing the severity of depressive symptoms and their progression. While MoCA has been validated locally and provides a quick cognitive screen, we recognize its limitations in patients with post-stroke aphasia. Future work should explore culturally validated alternatives like the Rowland Universal Dementia Assessment Scale (RUDAS) that minimize language dependency. Nevertheless, MoCA has been extensively used in Tanzanian population including post-stroke patient with reliable results.

The Barthel Index scale will be used to assess functionality and independency in daily living. Furthermore, we will also complement with the Modified Rankin Scale (mRS) to measure global disability and widely used stroke-specific disability measure and closely linked to depressive outcomes.

Given the nature of the study design and patients, the study has a risk of attrition, but while has a strong capacity to elucidate the causal relationship between dependent and outcome variables. The study is time-consuming and expensive, requiring close follow-up for accurate data collection. The study will not capture qualitative insights into stroke survivors’ lived experiences, particularly informal social support, spiritual coping, or community reintegration. A future mixed-methods study is warranted to identify modifiable psychosocial factors that may buffer post-stroke depression

The Dodoma University of Dodoma library, the study sites (Dodoma Regional Referral Hospital and Benjamin Mkapa Hospital), and a paper ready for submission in several peer-reviewed journals before publication will all receive the complete findings before they are published.
